# Knowledge, Attitudes, and Practices Regarding First Aid for Road Traffic Accidents Among the Romanian Population

**DOI:** 10.7759/cureus.97622

**Published:** 2025-11-24

**Authors:** Alice Beatrice Ifrim, Viviana Denisa Maro

**Affiliations:** 1 Department of Medico-Surgical and Complementary Sciences, Faculty of Medicine and Biological Sciences, Ștefan cel Mare University of Suceava, Suceava, ROU

**Keywords:** bystanders, first aid, medical education, road accident, romania

## Abstract

Introduction

Road traffic accidents represent a major public health issue worldwide, accounting for thousands of deaths every day. First aid is an essential, necessary, and mandatory intervention, comprising the immediate measures provided at the scene of the accident by a bystander, aimed at preventing further deterioration, assisting, and supporting the victims with minimal material and human resources until professional medical care becomes available. This study aimed to assess the knowledge, attitudes, and practices regarding first aid in the context of road traffic accidents among the Romanian population.

Materials and methods

An online questionnaire was developed and distributed to citizens via social networks. The questionnaire items addressed general and specific first aid questions.

Results

A total of 400 individuals responded to the questionnaire. More than half of the respondents report not having attended first aid courses (53.3%), and their knowledge level is medium (34.5%) or low (22.3%). For Section I, participants who had attended a first aid course achieved significantly higher scores on some items (e.g., first aid initiator: p=0.003, airway obstruction: p <0.001). For Section II, no significant differences were observed between groups in relation to rescuer safety (p=0.837) or victim relocation (p=0.025 - marginal significance). Additionally, a positive correlation was observed between the respondents' self-assessment of their level of first aid knowledge and the final score on the evaluation items (p=0.019; r^2^=0.30).

Discussions

Respondents who attended a first aid course scored, on average, approximately 3 points higher than those who did not. Although this result is statistically significant, it suggests a moderate effect of the training and highlights the influence of additional factors such as individual motivation, prior experience, teaching quality, and engagement level on the final knowledge outcome. Airway management was relatively well addressed; however, nearly three-quarters did not consider the jaw thrust technique or the recovery position. This highlights the need for a structured training course rather than relying solely on school activities or online resources. There appears to be a significant link between income and access to specialized first aid training. The positive attitude towards the importance of first aid knowledge and application is consistent with findings from other studies, including those conducted among the general public and medical students in Saudi Arabia, as well as the general population in the United Arab Emirates. Regarding Europe, there is a very limited number of studies on the provision of first aid in road accidents.

Conclusions

The study concluded that the general population has a moderate, yet insufficient, level of medical training for providing first aid in road traffic accidents despite a strongly positive attitude towards its importance and necessity.

## Introduction

Road traffic accidents are a major public health problem worldwide, leading to death, injury, lifelong suffering, and major economic losses. In 2022, at the United Nations High-Level Meeting on Global Road Safety, it was highlighted that road traffic accidents remain a major global public health concern, accounting for an estimated 1.35 million deaths each year [1]. More than 3.500 people worldwide lose their lives in road traffic accidents every day [2]. In addition, between 20 and 50 million people suffer non-fatal accidents, many of whom are affected by disabilities resulting from road traffic accidents [3]. Furthermore, road traffic injuries are considered a leading cause of death for people aged 5 to 29 [1,3]. At the same time, “as things stand, they are estimated to cause an additional 13 million deaths and 500 million injuries over the next decade and hinder sustainable development, particularly in low- and middle-income countries” [2].

Beyond physical injuries and disability, road traffic accidents also represent a major economic burden, ranging from hospitalization, rehabilitation, and reintegration into the workforce costs to legal and material expenses [1].

According to the latest reports, in 2024, a total of 4,235 road traffic accidents were recorded nationwide, resulting in 1,478 deaths and 3,247 serious injuries [4]. In such cases, the rapid intervention of a bystander can mean the difference between life and death [5]. First aid is an essential and necessary set of immediate actions carried out at the scene of the accident by the witness (spectator), aimed at preventing further harm, helping and supporting victims with minimal material and human resources, until professional medical care becomes available [6-11].

According to the European Resuscitation Council Guidelines, first aid is defined as “the initial care provided for an acute illness or injury to preserve life, alleviate suffering, prevent further illness or injury, and promote recovery,” while basic life support (BLS) focuses more on “initiating CPR (cardiopulmonary resuscitation) in any person who is unresponsive, with absent or abnormal breathing” [12]. The first hour after an injury is crucial for saving lives, and is known as the “golden hour.” Research indicates that high-quality prehospital and in-hospital care, especially during this time, can both prevent and reduce the risk of death. Research projects in six low- and middle-income countries found that prehospital care can reduce the risk of injury-related fatalities by 25% [13]. A 2018 meta-analysis review reported that bystander CPR improves survival in out-of-hospital cardiac arrest [14]. A study from the United States found that elderly patients who suffered out-of-hospital cardiac arrest had higher chances of surviving in the hospital if they received bystander CPR [15]. This underscores the crucial role of widespread basic life support (BLS) training in saving lives [6].

In most road traffic accident situations, common people are more likely to arrive at the scene of the accident before emergency services [16]; therefore, training the public in first aid and encouraging their active involvement can significantly reduce complications [10,13,17-19]. The bystander effect and the diffusion of responsibility are concepts first described in the 1960s, suggesting that bystanders are less likely to provide help when a large number of people are present. The literature emphasizes the importance of learning not only first aid skills, but also to understand how to overcome barriers to offering help [20]. Good Samaritan laws are inspired by a biblical parable, which defines a “Good Samaritan” as an individual who helps another person without prior obligation or expectation of reward [21]. In legal contexts, a Good Samaritan provides aid to a person who is injured or in distress*.* If the victim is unconscious or unresponsive, the rescuer may assume implied consent [21]. If the person is conscious and able to respond, the rescuer should ask for permission before offering help [21]. Good Samaritan laws protect against civil liability for ‘ordinary negligence,’ which refers to failure to act reasonably as a prudent person would in similar circumstances [21]. However, these laws do not protect against ‘gross negligence’ or intentional misconduct [21]. Gross negligence involves conscious and voluntary disregard of the need to act with care, creating a foreseeable risk of serious injury or harm to persons or property [21]. In Romania, individuals without formal medical training who voluntarily provide basic first aid, following instructions from a medical dispatch center or using basic first aid knowledge, and acting in good faith with the intention of preserving life or health, are not subject to criminal or civil liability, according to Law 95/2008, as amended, Title IV, Article 94.

Few studies on first aid knowledge have been published in Romania or even in Europe. Until today, only limited studies have assessed the knowledge, attitudes, and barriers to providing first aid among the general Romanian population.

This study aimed to assess knowledge, attitudes, and practices of first aid in road traffic accidents and to identify barriers that prevent citizens from offering help in emergencies, especially in road traffic accidents.

## Materials and methods

We developed an online questionnaire using Google Forms, based on a thorough literature review (Appendix 1) [6-8,22]. We distributed the survey link and a summary of the study objectives through various national social media platforms (WhatsApp, Facebook, Instagram, Discord). We conducted the study between March and May 2025. We applied no educational restrictions, aiming for a diverse sample representing the Romanian population. We did not collect personal identifiers (name, national ID), ensuring anonymity, and participants could withdraw at any time. By completing the questionnaire, participants consented to the use of their responses for scientific purposes.

Study design

The questionnaire was originally developed in English, consistent with the literature review, which had likewise been conducted in English. The questionnaire was subsequently translated into Romanian by the authors and reviewed by a certified translation specialist, given that the official language of the target group was Romanian. The questionnaire’s validity had already been tested and confirmed by other researchers prior to our study [6-8,22].

The questionnaire has four sections. The first section collected sociodemographic information, including age, sex, environment, county, marital status, presence of children, monthly income, education level, hours of sleep per night, possession of a driving license, driving experience, daily hours spent driving or traveling, presence of a first aid kit in the car, most frequently used transport, knowledge of the emergency number, awareness of traffic accidents in their city or country, previous experience as a witness to a traffic accident, whether they provided help, attendance of first aid courses or volunteer work in projects such as “Salvator din pasiune” and/or the Red Cross, and self-assessed first aid knowledge.

The second section focused on general first aid questions, containing 10 items, including: where to start first aid, who should provide it, moving the victim, airway obstruction, airway clearance maneuvers, victim positioning, correct ratio of chest compressions to ventilations, duration of resuscitation, first aid for fractures/bleeding, and interaction with conscious victims.

The third section covered first aid specific to traffic accidents and contains eight items: rescuer safety, moving victims, respiratory function, stopping bleeding, fracture immobilization, victim transport, and feeding and hydrating the victim.

The final section had a concluding character. It asked participants whether they would be interested in a first aid course, whether they consider first aid important, and whether it can save lives.

Course attendance was coded as a dichotomous variable (0 = no, 1 = yes). The dependent variable was the final score (continuous), allowing the use of linear regression to estimate the mean difference in performance between groups. We calculated knowledge scores by giving one point for each correct answer, except for the item addressing the initiation of first aid, for which two points were allocated to the correct response and one point to the partially correct response, with a maximum of 21 points for the second section and 8 points for the third section, totaling 29 possible points.

Sample size calculation

Based on previous studies, we used the Krejcie and Morgan formula to calculate the minimum required sample size:

$s = \frac{X^{2} N P (1 - P)}{d^{2}(N - 1) + X^{2} P (1 - P)}$

where S=required sample size, X2=tabulated chi-square value for 1 degree of freedom at the desired confidence level (3.841), N=population size, P=population proportion (assumed to be 50 to provide the largest sample size), and d=degree of precision expressed as a proportion (0.5) [23]. Krejcie and Morgan formulated predefined sample size tables based on the above formula. In the current study, an undetermined population was considered to determine the required sample size [23]. Therefore, the minimum sample size required was 384 for a 95% confidence level (CI) and a 5% margin of error.

Statistical analysis

All data obtained in this study were used strictly for research purposes, with complete confidentiality maintained; only the authors of this study had access to the data. The data were analyzed using Microsoft Excel 2016 (Microsoft Corporation, Redmond, USA), employing the t-test, Two-Sample Assuming Unequal Variances, and Regression tools. For each questionnaire item, the mean and standard deviation (SD) were calculated, and group comparisons were performed using the independent samples t-test. Statistical differences were considered significant for p-values <0.05.

## Results

The study sample included 400 respondents. Their sociodemographic data is presented in Table 1. Among the respondents, 302 (75.5%) were women and 98 (24.5%) were men. In terms of age, the majority of participants were in the 18-24 years category (61%), followed by 25-34 years (14.7%) and 35-44 years (11.5%). Most of them come from urban areas (59.1%), with Suceava County having the most respondents (62.2%). Regarding education, 41.8% had completed high school, 36% held a bachelor's degree, and 10.8% had a master's or doctoral degree. Almost half of the participants reported monthly incomes below 2000 RON (Romanian leu) (45.3%), while only 13.7% had over 6000 RON. Most respondents were single (72.2%) and had no children (76.8%), and 79.3% slept 6-8 hours per night. Most held a driver's license (69%), with 42% having less than 5 years of driving experience. Daily driving time was nearly equally divided between those who drove less than one hour (33.5%) and those who did not drive at all (31%). Most participants (81%) carried a first aid kit in their car, and only three respondents did not know the emergency number. Although the most used means of transport were personal car (54.8%) and public transport (53.8%), 40.2% of respondents declared that they did not know or did not care about the traffic accident situation, and over 85% considered the number of recent traffic accidents alarming. The majority of participants (71.3%) reported that they had never witnessed a traffic accident. Among the small proportion who had witnessed an accident but chose not to intervene, the main reason given was a lack of training (8%). More than half of the respondents reported not having attended first aid courses (53.3%), and their knowledge level was medium (34.5%) or low (22.3%). Regarding current knowledge acquisition, most participants stated they learned first aid during school or university classes (37.3%) or through social media (24.3%). Over 36% either did not update their knowledge or updated it only when they felt they had forgotten some information.

**Table 1:** Socio-demographic data *RON: Romanian leu, equivalent to 0.20 EUR, MR= Multiple Response

Parameter	Value	N (%)
Age	<18	9 (2.3)
	18-24	244 (61)
	25-34	59 (14.7)
	35-44	46 (11.5)
	45-54	33 (8.3)
	55-64	3 (0.7)
	>65	6 (1.5)
Gender	Female	302 (75.5)
	Male	98 (24.5)
Environment	Urban	237(59.25)
	Rural	163 (40.75)
County	Suceava	248 (62)
	Botoșani	28 (7)
	București	18 (4.5)
	Iași	17 (4.2)
	Other	89 (22.3)
Education	High school or less	181 (45.4)
	Undergraduate degree	144 (36)
	Postgraduate degree	43 (10.8)
	Postsecondary/professional degree	31 (7.7)
Income	<2000 RON	181 (45.3)
	2000-3000 RON	59 (14.7)
	3000-4000 RON	45 (11.3)
	4000-5000 RON	30 (7.5)
	5000-6000 RON	30 (7.5)
	>6000 RON	55 (13.7)
Marital status	Married	111 (27.8)
	Single	289 (72.2)
Parental status	Yes	93 (23.25)
	No	307 (76.75)
Sleep duration	<6 h	52 (13)
	6-8 h	317 (79.25)
	>8 h	31 (7.75)
Driving license	Yes	276 (69)
	No	84 (21)
	Next	40 (10)
Driving experience	Under 5 years	168 (42)
	Over 5 years	113 (25.25)
	No driving license	119 (29.75)
Hours driven/traveled daily	<1 h	134 (33.5)
	1-3 h	115 (28.75)
	>3 h	27 (6.75)
	No driving license	124 (31)
First aid kit in car	Yes	324 (81)
	No	37 (9.25)
	I don't know	39 (9.75)
Single emergency number	Yes	397 (99.3)
	No	3 (0.7)
Means of transport (MR)	Public transport	215 (53.8)
	Taxi/Uber/Bolt	64 (16)
	Personal car	219 (54.8)
	Motorcycle	4 (1)
	Bicycle	14 (3.5)
Number of accidents	Yes	239 (59.8)
	No	142 (35.5)
	Not interested	19 (4.7)
Accident witness	Yes	115 (28.7)
	No	285 (71.3)
Provided help	Yes	62 (15.5)
	No	63 (15.75)
	I don't know how to help	12 (3)
	I prefer not to get involved	5 (1.25)
	Not witnessed an accident	258 (64.5)
Barriers (MR)	Lack of training	32 (8)
	Lack of self-confidence	16 (4)
	Legal reasons	25 (6.3)
	Fear of providing first aid	12 (3)
	Lack of knowledge about first aid	20 (5)
	Not witnessed an accident	278 (69.5)
	Provided help	53 (13.3)
Specialized courses	Yes, recently (within the last year)	80 (20)
	Yes, more than 2 years ago	85 (21.2)
	No, but I would like to participate	213 (53.3)
	No, I am not interested	22 (5.5)
Specialized courses circumstances	Paid by employer	39 (9.75)
	Paid from own budget	31 (7.75)
	Other	97 (24.25)
	No specialized courses	233 (58.25)
Knowledge without courses (MR)	School/College	149 (37.3)
	Health Education Projects	90 (22.5)
	Public Information Campaigns	43 (10.8)
	Social Media	97 (24.3)
	No first aid knowledge	64 (16)
Refreshing knowledge (MR)	At 6 months	24 (6)
	At 1 year	24 (6)
	When they forget things	144 (36)
	When they are stressed out at work	24 (6.3)
	Doesn't refresh	146 (36.5)
	No first aid knowledge	61 (15.3)

For the second section (Appendix 2), the one on general first aid, the item with the most correct answers was “Where should first aid be initiated?” (97.75%), while the item with the fewest correct answers was “What is the correct compression-to-ventilation ratio in adult cardiopulmonary resuscitation (CPR)?” (52.25% incorrect).

Also, participants who had attended the first aid course achieved significantly higher scores (Table 2) on the following items: first aid initiator (t=3.615, p=0.0003), airway obstruction (t=3.622, =0.0003), airway clearance maneuvers (t=8.778, p <0.001), CPR ratio (t=8.709, p <0.001), CPR timing (t=7.879, p <0.001), fractures (t=5.154, p <0.001), and hemorrhages (t=5.449, p <0.001). Conversely, no significant differences were observed for the items regarding victim relocation (p=0.286) and positioning in airway obstruction (p=0.171).

**Table 2:** First aid training versus actual knowledge Course=1 → Attended a first aid course; Course=0 → Did not attend a first aid course; SD=Standard Deviation

Section 2	Question	Course=1 (mean ± SD)	Course=0 (mean ± SD)	t Stat	t Critic (two-tail)	p two-tail
	Place of first aid initiation	0.994 ± 0.08	0.966 ± 0.18	2.102	1.967	0.036
	First aid initiator	1.333 ± 0.62	1.102 ± 0.65	3.615	1.967	0.0003
	Victim relocation	1.024 ± 0.27	1.055 ± 0.31	-1.069	1.966	0.286
	Airway obstruction	2.09 ± 1.08	1.689 ± 1.11	3.622	1.967	0.0003
	Airway clearance maneuvers	1.49 ± 0.69	0.864 ± 0.73	8.778	1.966	< 0.001
	Positioning in airway obstruction	0.576 ± 0.5	0.506 ± 0.5	1.371	1.967	0.171
	CPR ratio	0.715 ± 0.45	0.311 ± 0.46	8.709	1.967	< 0.001
	CPR timing	1.685 ± 0.94	0.991 ± 0.76	7.879	1.968	< 0.001
	Fractures	0.933 ± 0.25	0.757 ± 0.43	5.154	1.966	< 0.001
	Hemorrhages	1.836 ± 0.81	1.391 ± 0.80	5.449	1.967	< 0.001
Section 3	Rescuer safety	0.957 ± 0.20	0.962 ± 0.19	-0.205	1.967	0.837
	Victim relocation	0.77 ± 0.42	0.668 ± 0.47	2.256	1.966	0.024
	Breathing assessment	1 ± 0	0.978 ± 0.14	2.255	1.97.	0.025
	Hemorrhage control	0.975 ± 0.15	0.974 ± 0.16	0.081	1.966	0.935
	Fracture immobilization	0.909 ± 0.28	0.787 ± 0.41	3.489	1.966	0.0005
	Victim transport to hospital	0.467 ± 0.50	0.455 ±0.50	0.223	1.966	0.823
	Victim hydration	0.212 ± 0.41	0.272 ± 0.45	-1.394	1.966	0.164
	Victim feeding	0.054 ± 0.22	0.060 ± 0.23	-0.213	1.966	0.83

In the third section (Appendix 3), the one on first aid items specific to road accidents, the most frequent correct answer was to the item “Do we make sure the victim is breathing properly?” (98.8%), followed by the one on stopping bleeding (97.5%) and rescuer safety (96%), and the item with the most incorrect answers was the one on transporting the victim to the hospital, where over 50% answered “no” (39.5%) or “I’m not sure” (14.5%).

Moreover, no significant differences were observed between groups in relation to rescuer safety (p=0.837), victim relocation (p=0.024 - marginal significance), breathing assessment (p=0.025 - marginal significance), hemorrhage control (p=0.935), fracture immobilization (p=0.0005), victim transport to hospital (p=0.823), victim hydration (p=0.164), and victim feeding (p=0.83) (Table 3).

**Table 3:** First aid training versus final score B=unstandardized coefficient; SE=standard error; β=standardized coefficient; CI=confidence interval; LL=lower limit; UL=upper limit.

Variable	B	SE	β	t	p	95% CI (LL, UL)
Intercept	14,79	0,25		59,76	<0.001	(14.30, 15.28)
First Aid Course	3,23	0,39	0,39	8,39	<0.001	(2.48, 3.99)

All the statistical differences observed in relation to the presence of a first aid course and the answer to the questions can be seen in Table 2.

For Section 1, participants who had attended the first aid course achieved significantly higher scores on the following items: first aid initiator (t=3.015, p=0.003), airway obstruction (t=3.622, p <0.001), airway clearance maneuvers (t=3.878, p <0.001), CPR ratio (t=8.009, p <0.001), CPR timing (t=7.879, p <0.001), fractures (t=3.151, p =0.002), and hemorrhages (t=5.449, p <0.001). Conversely, no significant differences were observed for the items regarding victim relocation (p=0.286) and positioning in airway obstruction (p=0.171).

With regard to Section 2, no significant differences were observed between groups in relation to rescuer safety (p=0.837), victim relocation (p=0.025 - marginal significance), breathing assessment (p=0.025 - marginal significance), hemorrhage control (p=0.029), fracture immobilization (p=0.001), victim transport to hospital (p=0.023), victim hydration (p=0.112), and victim feeding (p=0.83).

A linear regression analysis was also conducted to assess whether course attendance predicted the final score obtained in the evaluation (Table 3).

The model was significant, F(1, 398)=70.36, p <0.001, explaining approximately 15% of the variance in scores (R²=0.15).

## Discussion

In Romania, the number of accidents and related deaths decreased compared to last year, and the 2024 road risk indicators reached their lowest levels in the past 12 years. However, road traffic accidents remain a major public health and safety issue [4].

Contrary to expectations, only 28.7% (n=115) witnessed a road traffic accident, and only half of them (n=62) intervened to provide help. Consistent with the literature [10], the main reasons for not intervening (n=63) were lack of training (n=32), fear of legal repercussions (n=25), and lack of medical knowledge (n=20). These findings highlight the impact of poor medical education on first aid, since Romanian law does not penalize individuals who voluntarily help victims in critical situations. Only 32.75% of the participants reported good and very good knowledge of first aid, compared to 67.25% who declared medium, low, or no knowledge. However, the level of knowledge tends to be similar across both groups, with few extremes. The main sources of knowledge were school or university activities (n=149), extracurricular activities (n=90), and social media (n=97). Public information campaigns had the lowest impact, with only 43 respondents identifying them as a source of first aid knowledge.

A positive correlation was identified (p=0.0003) between participation in a specialized first aid course and correctly identifying the bystander as the person responsible for initiating first aid, as stated in guidelines and the literature. In contrast, only 225 out of 400 respondents recognized "absence of breathing" as a sign of airway compromise, although this should be considered common knowledge regarding the critical condition of an individual.

The data show statistically significant differences in the key aspects of immediate survival: cardiopulmonary resuscitation (CPR) performance and duration, airway management, and hemorrhage control. Very high t-values (p <0.001) confirm the evident impact of the first aid course on skills in critical situations.

There are a few situations where no differences were observed between participants who attended the first aid course and those who did not: rescuer safety (p=0.837) - surprisingly, given that it is a fundamental principle. This result may suggest that the topic was not sufficiently emphasized during the course, or that both groups share similar (possibly intuitive) perceptions; hydration and feeding of the victim (p >0.05) - no differences were observed here, likely because these aspects are secondary and not part of the critical maneuvers, while courses focus primarily on immediate emergencies.

Regression analysis indicated that course attendance accounted for only 15% of the variance in final performance (R²=0.15) and was associated with a significant increase in the final score (β=3.23, SE=0.39, t(398)=8.39, p <0.001, 95% CI [2.48,3.99]). Respondents who attended the course scored, on average, approximately 3 points higher than those who did not. Although this result is statistically significant, it suggests a moderate effect of the training and highlights the influence of additional factors such as individual motivation, prior experience, teaching quality, and engagement level on the final knowledge outcome. Moreover, relatively high standard deviations for certain variables (e.g., fractures, hemorrhages) indicate unequal knowledge acquisition within the trained group, pointing to a lack of uniformity in the course’s effectiveness.

Regarding victim safety, although most respondents (n=312) correctly ticked that moving the victim is appropriate only in the case of imminent danger, only 105 were aware of the essential rule of avoiding unnecessary movement. The difference was not significant (p=0.291 for Section 2; p=0.025 for Section 3, though marginal). Victim relocation should be a central aspect, yet the values do not indicate a clear advantage for those who attended the first aid course.

Airway management was relatively well addressed, with the majority recognizing the head-tilt chin-lift maneuver; however, nearly three-quarters did not consider the jaw thrust technique or the recovery position. This highlights the need for a structured training course rather than relying solely on school activities or online resources. Surprisingly, fewer than half of the respondents knew the correct compression-to-ventilation ratio, a key element in performing cardiopulmonary resuscitation. In terms of bleeding control, basic knowledge of direct pressure and bandaging was adequate, but only half considered the use of a tourniquet, and just one-quarter recognized the importance of elevating the injured limb above heart level. 

Given that over 87% of participants considered first aid to be important, yet 53.3% had not attended a first aid course, and nearly half reported a monthly income below 2000 RON, there appears to be a significant link between income and access to specialized first aid training. This positive attitude towards the importance of first aid knowledge and application is consistent with findings from other studies, including those conducted among the general public and medical students in Saudi Arabia [24,25] as well as the general population in the United Arab Emirates [26]. Regarding Europe, there is a very limited number of studies on the provision of first aid in road accidents, representing a gap in contemporary medical research and practice. The literature highlights only a single article that points to the same persistent deficiencies [27].

The data presented above indicate that participants possess a general set of first aid knowledge; however, this knowledge often proves to be fragmented and insufficiently structured. Although most respondents expressed a theoretical willingness to intervene, their confidence in their own actions remained low, which may significantly limit the effectiveness of real-life interventions. Therefore, additional educational initiatives are needed-structured and recurrent programs that not only consolidate existing knowledge but also strengthen practical skills and build the confidence required to correctly apply first aid measures in real scenarios, ultimately improving outcomes for victims of road traffic accidents and other emergencies. Unfortunately, mandatory first aid courses are not provided in Romanian schools or universities. Moreover, although Romania’s theoretical driving test includes questions related to first aid, no practical training is offered. These findings align with the challenges observed in practice.

Strengths and limitations

This study has some limitations. First, the uneven distribution of participants across counties may have influenced the results. Second, disseminating the questionnaire primarily through social media restricted access for certain population groups and limited overall participation, as the general population tends to show low responsiveness to such surveys. Despite these challenges, the study successfully captures a representative category relevant to the educational needs in Romania. The findings clearly highlight gaps in general medical knowledge related to road traffic accidents. Therefore, the study not only addresses a critical gap in the existing literature but also emphasizes the urgent need for initiatives aimed at strengthening first aid training in Romania.

Future directions

Future studies should include larger and more diverse populations to provide a clearer picture of knowledge levels and responses in first aid. Research should also address multiple aspects of first aid, not only theoretical knowledge but also practical skills. Road traffic accidents remain a major public health problem. Strengthening first aid training can improve survival, reduce the burden on the healthcare system, and indirectly benefit the economy.

## Conclusions

The findings of this study indicate that the general population has a moderate but insufficient level of preparedness to provide first aid in road traffic accidents, despite a strong positive attitude toward its importance and necessity. First aid courses significantly improve essential life-saving knowledge; however, their effect is moderate and requires practical engagement and real-world experience. The data indicate that formal training is effective but incomplete and uneven. For a meaningful societal impact, courses should be expanded, standardized, and supplemented with repeated practical simulations

To improve outcomes in emergencies, we strongly recommend the implementation of structured and regular training courses, delivered by qualified professionals, to reach as many citizens as possible.

The findings support the recommendation that mandatory practical first aid modules should be introduced for all candidates applying for a driving license, as well as compulsory first aid training in schools and universities. This measure is strongly supported by the fact that most participants identified a lack of training, insufficient knowledge, and fear of performing first aid as the main barriers that prevent them from intervening when needed.

Furthermore, considering the link between economic status and attendance at specialized courses, it is essential to ensure that these courses are accessible to all socio-economic groups, particularly those with low incomes. Through these approaches, the community can be encouraged to adopt a more open and proactive attitude, prepared to respond effectively in emergency situations.

## Appendices

Appendix 1: Questionnaire

You are invited to participate in a research study on road traffic accidents and the level of knowledge regarding first aid measures. The study is conducted by two registered nurses, Master’s students at the Faculty of Medicine and Biological Sciences, “Ștefan cel Mare” University of Suceava. Your personal data provided in this informed consent will be kept strictly confidential and will only be accessible to the research team. The data collected through the questionnaire will be analyzed using statistical methods that do not allow the disclosure of any information regarding your identity or opinions. In the event of publication, no individual information will be revealed. Your participation in this study is voluntary.

By completing this form, you consent to the collection and processing of your personal data in accordance with Regulation (EU) 2016/679 (GDPR), for the purpose of organizing and conducting this study. Your data will be used exclusively for research purposes and will not be shared with third parties without your prior consent. You have the right to access, rectify, delete, or request the restriction of processing of your personal data, in accordance with applicable legislation. The estimated time to complete the questionnaire is approximately 5-7 minutes.

**Table 4:** Questionnaire Form *correct answer; **partially correct answer; MR: multiple responses

Question	Answer Options
First section: Socio-demographic data	
1. What is your age?	☐ under 18 years
	☐ 18-24 years
	☐ 25-34 years
	☐ 35-44 years
	☐ 45-54 years
	☐ 55-64 years
	☐ over 65 years
2. Place of residence	☐ Urban
	☐ Rural
3. What is your gender?	☐ Female
	☐ Male
4. Your county of residence	
5. What is the highest level of education you have completed?	☐ No formal education
	☐ Primary education
	☐ Secondary education (middle school)
	☐ High school education
	☐ Post-secondary / vocational school
	☐ University degree (Bachelor’s)
	☐ Postgraduate studies (Master’s/Doctorate)
6. What is your monthly income level?	☐ less than 2000 RON
	☐ 2000-3000 RON
	☐ 3000-4000 RON
	☐ 4000-5000 RON
	☐ 5000-6000 RON
	☐ more than 6000 RON
7. What is your marital status?	☐ Married
	☐ Single
8. Do you have children?	☐ Yes
	☐ No
9. How many hours do you sleep per night?	☐ less than 6 hours
	☐ 6-8 hours
	☐ more than 8 hours
10. Do you hold a driver’s license?	☐ Yes
	☐ No
	☐ In progress
11. What is your driving experience?	☐ less than 5 years
	☐ 5-10 years
	☐ 10-15 years
	☐ 15-20 years
	☐ over 20 years
	☐ I do not have a driver’s license
12. How many hours do you drive/travel daily?	☐ less than 1 hour
	☐ 1-3 hours
	☐ more than 3 hours
	☐ I do not drive/I do not have a driver’s license
13. Do you have a first aid kit in your personal/family car?	☐ Yes
	☐ No
	☐ I don’t know
14. Do you know the emergency number to call in case of need?	☐ Yes
	☐ No
15. What is the means of transport you use most often? (you may select multiple options)	☐ Public transportation (bus, minibus, train)
	☐ Taxi/Uber/Bolt
	☐ Personal car
	☐ Motorcycle
	☐ Bicycle
16. Are you aware of the frequency/number of accidents in your city/country?	☐ Yes
	☐ No
	☐ I am not interested
17. Do you consider the number of road accidents in recent times alarming?	☐ Yes
	☐ No
	☐ I am not interested
18. Have you ever witnessed a road accident?	☐ Yes
	☐ No
19. If you answered YES, did you intervene to provide help?	☐ Yes
	☐ No
	☐ I don’t know how to help
	☐ I prefer not to get involved
	☐ I have not witnessed a road accident
20. If you did not intervene, what was the reason? (you may select multiple options)	☐ Lack of first aid training/practice
	☐ Lack of self-confidence
	☐ Legal concerns
	☐ Fear of providing first aid
	☐ Lack of knowledge about first aid
	☐ I have not witnessed a road accident
	☐ I did provide help
21. Have you ever attended a specialized first aid course?	☐ Yes, recently (within the last year)
	☐ Yes, but more than 2 years ago
	☐ No, but I would like to participate
	☐ No, I am not interested
22. If you answered YES, under what circumstances did you attend the specialized first aid course?	☐ Specialized training at the workplace, funded by the employer
	☐ Specialized training on my own initiative, self-funded
	☐ Other
	☐ I have not attended a specialized first aid course
23. Have you been or are you a volunteer in first aid projects (e.g., “Rescuer by Passion” , Red Cross, etc.)?	☐ Yes
	☐ No
24. How would you assess your first aid knowledge?	☐ Very good
	☐ Good
	☐ Average
	☐ Poor
	☐ I have no knowledge of first aid
25. If you have not attended a specialized first aid course but you possess the necessary knowledge, where did you acquire it? (you may select multiple options)	☐ Learned during school/university classes
	☐ Learned at school within health education projects (e.g., “Different School Week,” “Green School”)
	☐ Learned through public awareness campaigns
	☐ Learned from social media (YouTube, Instagram, Facebook, TikTok, etc.)
	☐ I have no first aid knowledge
	☐ I have attended a specialized first aid course
26. How often do you update your knowledge of first aid? (you may select multiple options)	☐ Every 6 months
	☐ Every year
	☐ Whenever I feel I have forgotten some things
	☐ When required by my workplace (if applicable)
	☐ I do not usually update my learned notions
	☐ I have no knowledge of first aid
Second section: First aid - general	
27. Where should first aid be initiated?	☐ At the accident site*
	☐ As soon as the victim arrives at the hospital
	☐ I don’t know
28. Who should initiate first aid?	☐ A bystander*
	☐ Only those who know airway clearance maneuvers**
	☐ Only medical personnel
	☐ I don’t know
29. Under what circumstances is it acceptable to move a victim from the accident site? (you may select multiple options)	☐ If there is an immediate danger, such as a fire*
	☐ If the victim has a minor injury
	☐ If the victim requests to be moved
	☐ It is never recommended to move a victim*
30. What are the signs of airway obstruction? (you may select multiple options)	☐ Rapid breathing*
	☐ Noisy breathing*
	☐ Labored breathing*
	☐ Not breathing*
	☐ I don’t know
31. Which of the following are airway clearance maneuvers? (you may select multiple options)	☐ Jaw thrust*
	☐ Head tilt-chin lift*
	☐ Recovery position*
	☐ I don’t know
32. How do you position a victim with a compromised airway?	☐ Place the victim on their side*
	☐ Place the victim on their back
	☐ I don’t know
33. What is the correct compression-to-ventilation ratio in adult cardiopulmonary resuscitation (CPR)?	☐ 15 compressions - 2 ventilations
	☐ 30 compressions - 2 ventilations*
	☐ 10 compressions - 1 ventilation
	☐ I don’t know
34. How long should resuscitation maneuvers continue? (you may select multiple options)	☐ Until medical teams arrive*
	☐ Until the rescuer is exhausted*
	☐ Until pulse returns*
	☐ No longer than 20 minutes, to avoid rib fractures
	☐ I don’t know
35. How do you provide first aid in the case of fractures?	☐ Apply a splint*
	☐ Leave it free until the victim reaches the hospital
	☐ I don’t know
36. What is the first aid for hemorrhage (bleeding)? (you may select multiple options)	☐ Apply pressure and a pressure bandage on the bleeding wound*
	☐ Clean with rubbing alcohol
	☐ Apply a tourniquet*
	☐ Raise the injured limb above heart level*
	☐ I don’t know
Third section: First aid - road accidents	
39. Should the rescuer’s safety be prioritized (to avoid creating more victims)?	☐ Yes*
	☐ No
	☐ I am not sure
40. Should victims be moved from the accident site if the situation requires it?	☐ Yes*
	☐ No
	☐ I am not sure
41. Should we ensure that the victim is breathing properly?	☐ Yes*
	☐ No
	☐ I am not sure
42. Should we stop the bleeding?	☐ Yes*
	☐ No
	☐ I am not sure
43. Should we immobilize fractures?	☐ Yes*
	☐ No
	☐ I am not sure
44. Should we transport the victim to the hospital?	☐ Yes*
	☐ No
	☐ I am not sure
45. Should we give the victim water/fluids?	☐ Yes
	☐ No*
	☐ I am not sure
46. Should we give the victim food?	☐ Yes
	☐ No*
	☐ I am not sure
Final section Thank you for your participation!	
After completing this questionnaire, would you be interested in attending a first aid course?	☐ Yes
	☐ No
	☐ I don’t know yet
	☐ I have already attended a first aid course
Do you believe that first aid is important and can save lives?	☐ Strongly disagree
	☐ Disagree
	☐ Neutral
	☐ Agree
	☐ Strongly agree

Appendix 2: Section 2 - Response frequencies for general first aid items

**Table 5:** Section 2: Response frequencies for general first aid items *correct answer; MR: multiple responses

Question	Answer Options	N (%)
Where should first aid be initiated?	At the accident site^*^	391 (97.75)
	As soon as the victim arrives at the hospital	2 (0.5)
	I don’t know	7 (1.75)
Who should initiate first aid?	A bystander^*^	130 (32.5)
	Only those who know airway clearance maneuvers^*^	219 (54.75)
	Only medical personnel	35 (8.75)
	I don’t know	16 (4)
Under what circumstances is it acceptable to move a victim from the accident site? (MR)	If there is an immediate danger, such as a fire^*^	312 (78)
	If the victim has a minor injury	75 (18.8)
	If the victim requests to be moved	11 (2.8)
	It is never recommended to move a victim^*^	105 (26.3)
What are the signs of airway obstruction? (MR)	Rapid breathing^*^	63 (15.8)
	Noisy breathing^*^	145 (36.3)
	Labored breathing^*^	271 (67.8)
	Not breathing^*^	263 (65.8)
	I don’t know	33 (8.3)
Which of the following are airway clearance maneuvers? (MR)	Jaw thrust^*^	79 (19.8)
	Head tilt–chin lift^*^	253 (63.2)
	Recovery position^*^	117 (29.3)
	I don’t know	83 (20.8)
How do you position a victim with a compromised airway?	Place the victim on their side^*^	214 (53.5)
	Place the victim on their back	119 (29.75)
	I don’t know	67 (16.75)
What is the correct compression-to-ventilation ratio in adult cardiopulmonary resuscitation (CPR)?	15 compressions – 2 ventilations	48 (12)
	30 compressions – 2 ventilations^*^	191 (47.75)
	10 compressions – 1 ventilation	35 (8.75)
	I don’t know	126 (31.5)
How long should resuscitation maneuvers continue? (MR)	Until medical teams arrive^*^	259 (64.8)
	Until the rescuer is exhausted^*^	78 (19.5)
	Until pulse returns*	174 (43.5)
	No longer than 20 minutes, to avoid rib fractures	39 (9.8)
	I don’t know	47 (11.8)
How do you provide first aid in the case of fractures?	Apply a splint^*^	332 (83)
	Leave it free until the victim reaches the hospital	16 (4)
	I don’t know	52 (13)
What is the first aid for hemorrhage (bleeding)? (MR)	Apply pressure and a pressure bandage on the bleeding wound^*^	308 (77)
	Clean with rubbing alcohol	18 (4.5)
	Apply a tourniquet^*^	217 (54.3)
	Raise the injured limb above heart level^*^	105 (26.3)
	I don’t know	22 (5.5)

Appendix 3: Section 3 - Response frequencies for first aid items specific to road accidents

**Table 6:** Section 3 - Response frequencies for first aid items specific to road accidents *correct answer

Question	Answer Options	N (%)
Should the rescuer’s safety be prioritized (to avoid creating more victims)?	Yes^*^	384 (96)
	No	4 (1)
	I am not sure	12 (3)
Should victims be moved from the accident site if the situation requires it?	Yes^*^	284 (71)
	No	51 (12.75)
	I am not sure	65 (16.25)
Should we ensure that the victim is breathing properly?	Yes^* ^	395 (98.8)
	No	1 (0.2)
	I am not sure	4 (1)
Should we stop the bleeding?	Yes^*^	390 (97.5)
	No	3 (0.75)
	I am not sure	7 (1.75)
Should we immobilize fractures?	Yes^*^	335 (83.75)
	No	18 (4.5)
	I am not sure	47 (11.75)
Should we transport the victim to the hospital?	Yes^*^	184 (46)
	No	150 (39.5)
	I am not sure	58 (14.5)
Should we give the victim water/fluids?	Yes	99 (24.75)
	No^*^	189 (47.25)
	I am not sure	112 (28)
Should we give the victim food?	Yes	23 (5.75)
	No^*^	318 (79.5)
	I am not sure	59 (14.75)
